# PFΔ*Screen* — an open-source tool for automated PFAS feature prioritization in non-target HRMS data

**DOI:** 10.1007/s00216-023-05070-2

**Published:** 2023-11-30

**Authors:** Jonathan Zweigle, Boris Bugsel, Joel Fabregat-Palau, Christian Zwiener

**Affiliations:** 1 Environmental Analytical Chemistry, Department of Geosciences, University of Tübingen, Schnarrenbergstraße 94-96, 72076 Tübingen, Germany; 2 Hydrogeochemistry, Department of Geosciences, University of Tübingen, Schnarrenbergstraße 94-96, 72076 Tübingen, Germany

**Keywords:** PFAS, Non-target screening, Feature prioritization, HRMS, Open-source software, Mass defect, MD/C-m/C, KMD

## Abstract

Per- and polyfluoroalkyl substances (PFAS) are a huge group of anthropogenic chemicals with unique properties that are used in countless products and applications. Due to the high stability of their C-F bonds, PFAS or their transformation products (TPs) are persistent in the environment, leading to ubiquitous detection in various samples worldwide. Since PFAS are industrial chemicals, the availability of authentic PFAS reference standards is limited, making non-target screening (NTS) approaches based on high-resolution mass spectrometry (HRMS) necessary for a more comprehensive characterization. NTS usually is a time-consuming process, since only a small fraction of the detected chemicals can be identified. Therefore, efficient prioritization of relevant HRMS signals is one of the most crucial steps. We developed PFΔ*Screen*, a Python-based open-source tool with a simple graphical user interface (GUI) to perform efficient feature prioritization using several PFAS-specific techniques such as the highly promising MD/C-m/C approach, Kendrick mass defect analysis, diagnostic fragments (MS^2^), fragment mass differences (MS^2^), and suspect screening. Feature detection from vendor-independent MS raw data (mzML, data-dependent acquisition) is performed via pyOpenMS (or custom feature lists) with subsequent calculations for prioritization and identification of PFAS in both HPLC- and GC-HRMS data. The PFΔ*Screen* workflow is presented on four PFAS-contaminated agricultural soil samples from south-western Germany. Over 15 classes of PFAS (more than 80 single compounds with several isomers) could be identified, including four novel classes, potentially TPs of the precursors fluorotelomer mercapto alkyl phosphates (FTMAPs). PFΔ*Screen* can be used within the Python environment and is easily automatically installable and executable on Windows. Its source code is freely available on GitHub (https://github.com/JonZwe/PFAScreen).

**Graphical abstract:**

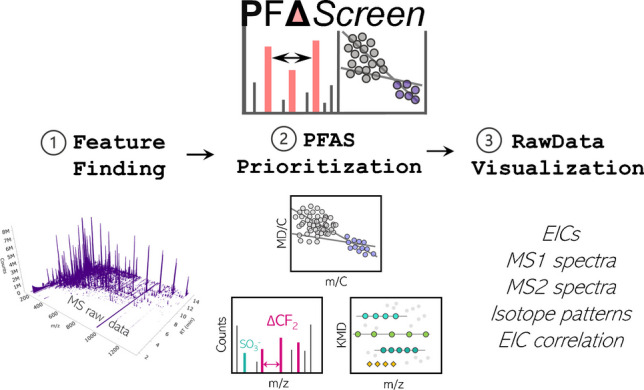

**Supplementary Information:**

The online version contains supplementary material available at 10.1007/s00216-023-05070-2.

## Introduction

Per- and polyfluoroalkyl substances (PFAS) are a large group of anthropogenic chemicals characterized by containing multiple C-F bonds [1, 2]. Due to their unique properties, they are used in a wide array of daily products and different industrial applications [3]. Their high chemical resistance and water and oil repellency lead to the production of PFAS with a variety of different chemistries [4]. Due to the high stability of C-F bonds, the perfluoroalkyl chains of PFAS exhibit an intrinsic persistence that leads to a worldwide distribution of PFAS and their terminal transformation products (TPs) such as perfluoroalkyl acids (PFAAs) which were extensively produced and used in the past [5–8]. Nowadays, the number of known PFAS ranges from thousands to millions, depending on the definition and source of information. According to the updated OECD definition, all chemicals containing a CF_3_ or isolated CF_2_ group are considered PFAS, which has increased the number of PFAS considerably [9, 10]. Global regulatory efforts restricted the production of selected longer-chain PFAAs such as perfluorooctanoic acid (PFOA) and perfluorooctanesulfonic acid (PFOS) due to their persistence, bioaccumulation potential, and adverse effects on humans and the environment [11]. This resulted in the production of replacement compounds of rather similar persistence, increasing the number of different PFAS on the global market that are also eventually emitted into the environment [12]. Therefore, PFAS are considered to be regulated as a chemical class in the European Union in the future [13].

Several studies have shown that considerable fractions of organically bound fluorine (e.g., extractable organic fluorine) in environmental and human samples cannot be explained sufficiently by routinely analyzed PFAS (target screening), which usually include less than 50 analytes [14–17]. Since almost no fluorinated organic compounds occur naturally, unknown fractions of organically bound fluorine are clear indications of anthropogenic chemicals [18].

Due to the sheer number of different PFAS that transform into an even larger number of unknown TPs, a comprehensive use of authentic reference standards is usually not possible and most likely will not be soon [19, 20]. The fact that PFAS are industrial chemicals that often underlie the trade secrets even complicates the availability of standards. Therefore, non-target screening (NTS) based on high-resolution mass spectrometry (HRMS) is necessary for a more comprehensive characterization of PFAS [21, 22]. Several studies have shown that target analysis is insufficient to capture PFAS present in complex samples, which can easily result in the overlooking of important compounds even when present in high concentrations [23]. NTS approaches led to the identification of more than 750 novel PFAS in various samples in the past worldwide, showing their high relevance in analytical approaches [22, 24]. Since NTS is typically a time-consuming and often partially manual process, efficient prioritization techniques are needed to separate detected matrix components from the analytes of interest (often a data reduction from  ~ 5000 detected compounds to 10–100 identified analytes or even less) [25].

**Fig. 1:**
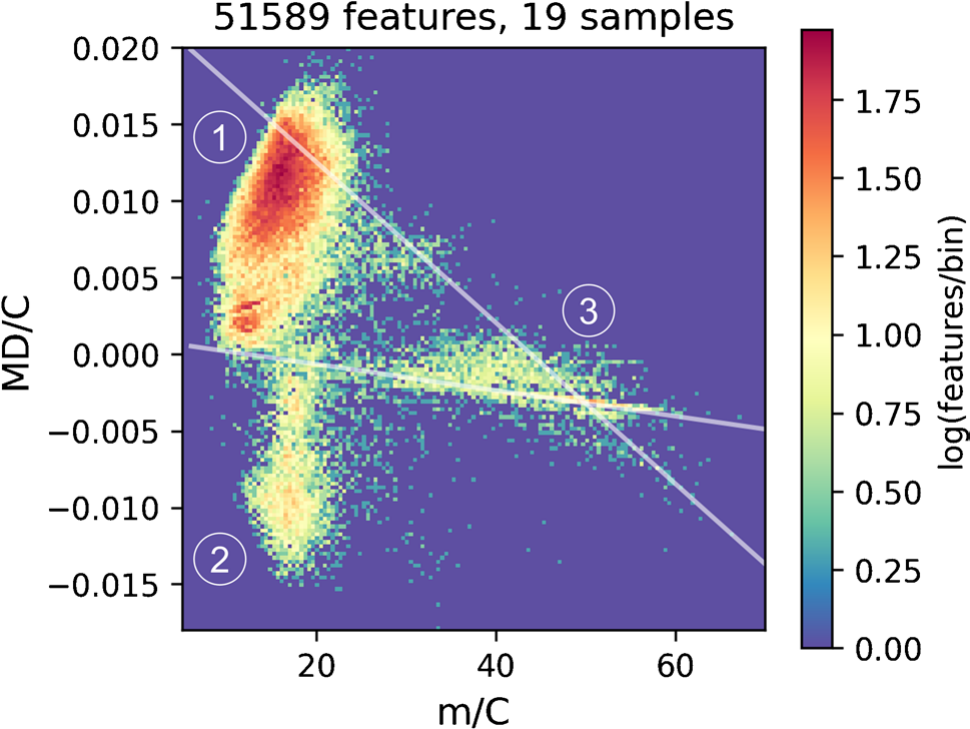
2D histogram of the number of compounds (log scale) (compound density) in the MD/C-m/C plot of 19 measured samples used from several paper and soil extracts, standards, and blanks (19 samples with 51,589 features from [23, 31, 32]). Hydrocarbon features are located usually below m/C of 25 with a clearly positive MD/C (position 1), while at a certain C number the MD exceeds + 0.5 Da yielding a position of a mathematical negative MD/C (position 2). Highly fluorinated compounds or compounds with other heavy heteroatoms are strongly shifted to higher m/C values (position 3). It becomes obvious that even with these high numbers of features in several samples from several different matrices, potential PFAS features with a certain fraction of fluorine within the molecule are efficiently separated from most matrix components. The gray lines mark the CH_x_F_2-x_-line (0 ≤ *x* ≤ 2) and the CF_x_-line (0 ≤ *x* ≤ 2) (for details on the MD/C-m/C plot, see Zweigle et al. [26])

The intrinsic properties of PFAS (with a certain fluorine percentage) allow the use of several techniques for their prioritization [21, 26]: The chemical mass defect (MD) of PFAS is typically lower (MD_F_ =  − 0.0016 Da) than the one of hydrocarbons (MD_H_ =  + 0.0078 Da) and has been used to remove detected features outside a predefined MD range (e.g.,  − 0.25 to  + 0.1 Da) [27–29]. However, this range is not fixed, and depending on the structure, it is important to know that hydrocarbons of higher mass that exceed a MD of  + 0.75 Da can also fall into the same range. Similarly, polyfluorinated PFAS with a high H content may bear a positive MD exceeding + 0.1 Da. Recently, a promising approach based on the MD normalized to the carbon number (MD/C) vs. the mass normalized to the carbon number (m/C) was proposed to separate PFAS much more efficiently from other hydrocarbon features in HRMS data which was further systematically evaluated for  ~ 200,000 PFAS from chemical databases [26, 30]. The carbon number can be easily estimated for all HRMS features by using the relative abundance of the M+1 isotope (^13^C). PFAS have a much higher m/C when their mass is dominated by fluorine (e.g., m/C ~ 50), while hydrocarbons of similar mass are dominated by carbon (m/C ~ 14), allowing a convenient separation. Details on the MD/C-m/C approach are summarized in Zweigle et al. [26]. Especially, the m/C dimension can be used to remove large fractions of non-PFAS features when applied appropriately. This is illustrated in Fig. 1 with a 2D histogram of the MD/C-m/C locations of over 50,000 features from previous HRMS measurements of PFAS-contaminated soils and grease-repelling papers, where a clear separation of potentially highly fluorinated compounds is observed (region around m/C ≈ 40, MD/C =  − 0.002). It is important to note, however, that the MD/C-m/C separation works better the higher the percentage of fluorine in a molecule is, with an accordingly higher F/C and a lower H/F ratio [26]. Like the MD, the MD/C-m/C approach cannot separate, for instance, hydrocarbons with one or two CF_3_ groups from other hydrocarbons.

Besides the MD and MD/C-m/C approach, the Kendrick mass defect (KMD) analysis to detect homologous series of PFAS (e.g., with CF_2_ or CF_2_O as repeating units) is of great relevance since it allows the grouping of structurally related PFAS, simplifying their identification [27, 33]. In the MS^2^ data, lists of PFAS-specific diagnostic fragments (DFs) as well as fragment mass differences and neutral losses can be used to prioritize fragmentation spectra [28, 31, 34]. These techniques are often combined with suspect screening by matching accurate mass (or further evidence) with PFAS lists [22, 35].

KMD, DFs, fragment mass differences, and especially suspect screening with large lists (e.g., PFASMASTER, gathering over 12,000 compounds [36]) in combination with complex samples (thousands of features) are prone to a high number of false-positive detections (depending on mass tolerance) that often need to be excluded manually, which is a time-consuming process. Even with extremely high mass resolution, naturally occurring compounds can still mimic certain PFAS-specific repeating units such as CF_2_, complicating KMD analysis and making retention time shifts a necessary criterion [37]. Therefore, if the number of features can be preliminarily reduced by the MD/C-m/C approach before applying those techniques, a faster and more accurate NTS workflow can be performed, decreasing both computational and manual effort regarding the further inspection of the features. Although many of the above discussed PFAS-specific techniques for prioritization and identification are applied, they are often not performed in a systematic way using open-source software [38]. Therefore, it is important to combine the data processing in a more systematic step-wise procedure.

To facilitate the non-targeted screening of PFAS in complex samples, we developed PFΔ*Screen*, an open-source Python-based software tool with a simple graphical user interface (GUI) that combines the discussed techniques to efficiently prioritize PFAS in LC- or GC-HRMS data acquired with electrospray (ESI) or atmospheric pressure chemical ionization (APCI). PFΔ*Screen* can be applied vendor-independently either on mass spectrometric raw data (mzML, automated feature finding via pyOpenMS) or on custom feature lists (external feature finding by other software tools). The PFΔ*Screen* workflow presented here is then applied to four PFAS-contaminated agricultural soil extracts from south-western Germany (Rastatt case [27, 39]), where several PFAS classes, including novel PFAS, were identified. The advantages of the combined workflow are discussed in detail. The source code is available via GitHub and can be easily automatically installed and executed via batch files on Windows within the Python environment. The Python source code can also be executed on other operating systems within the Python environment (without automatic installation).

## Materials and methods

### PFΔ*Screen* workflow

**Fig. 2:**
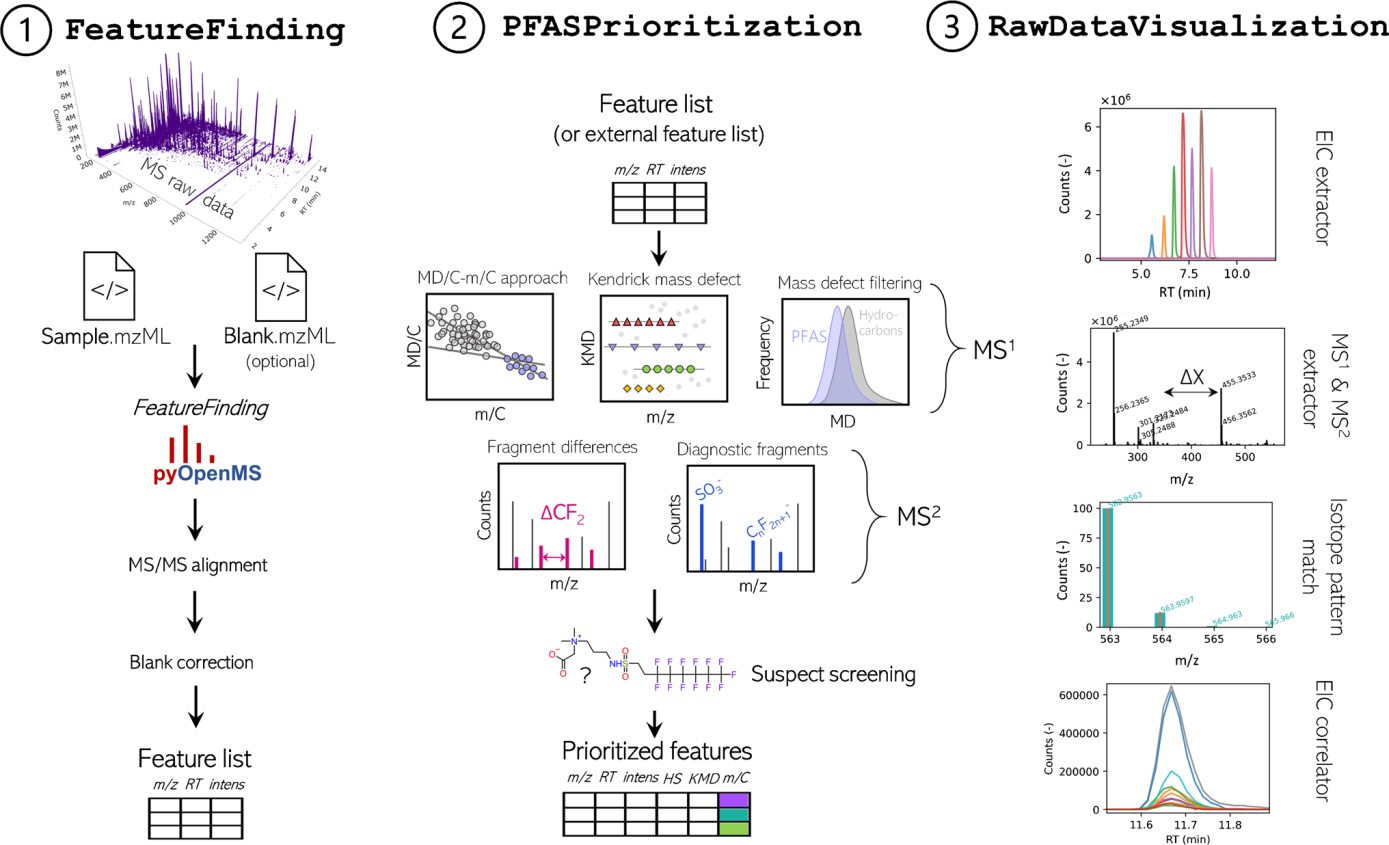
Schematic overview of the PFΔ*Screen* workflow in the structure of the GUI (Fig. S1). The FeatureFinding tab (1) allows detection of feature via pyOpenMS in MS raw data followed by MS^2^ alignment and blank correction resulting in a feature list for a sample of interest. PFAS feature prioritization (2) includes techniques such as the MD/C-m/C approach, KMD analysis, fragment matching, and fragment mass differences which generates a strongly reduced feature list of potential PFAS. The data from this list can be visualized and verified by the RawDataVisualization tool (3) together with other output files such as interactive HTML plots which allow efficient NTS (Fig. S3–S5)

PFΔ*Screen* is a fully automated tool for detection and prioritization of potential PFAS features (LC- or GC-HRMS with ESI or APCI source) in raw mass spectrometric data written in Python (3.9.13) (Fig. 2). PFΔ*Screen* is structured in several individual Python functions that are executed from one main file that allows data and parameter input via a simple GUI programmed with the tkinter library (Fig. S1). It can easily be automatically installed and executed on Windows using batch files. Detailed instructions on installation and functionality are provided in the SI. Input MS raw data can be converted vendor-independently from data-dependent acquisition (ddMS^2^) files into the mzML data format (.mzML) by using the MSConvert software from ProteoWizard [40, 41]. Only mzML files with centroided spectra and one collision energy (CE) should be used. If profile data was acquired and MS^2^ spectra from several different CEs per precursor m/z are present, the peak picking (for centroiding) and subset functions (to keep only one desired CE) from MSConvert can be used to generate the correct mzML input files.

In the following, the three main functionalities of PFΔ*Screen* are explained in the same order as they can be executed in the GUI (Fig. 2 and Fig. S1).

#### FeatureFinding

The first step usually performed in NTS is detection of features in the MS raw data characterized by chromatographic peak shapes of coeluting isotopes, resulting in a list of m/z, retention time (RT), and peak area. This task is performed with pyOpenMS, a Python interface to the C++ OpenMS library [42–46]. For feature detection, the FeatureFinderMetabo algorithm is used, which is designed for metabolites and small molecules [47–49]. Three parameters (mass error (ppm), intensity threshold, and an isotope model for more accurate detection of coeluting isotopologues) can be specified. The most important parameter is the intensity threshold, which is highly dependent on the instrument used, sample, and the underlying NTS question. After feature finding in the MS^1^ data, MS^2^ spectra can be aligned to their respective precursors by specifying an m/z and RT tolerance. Only one unique MS^2^ spectrum with the highest precursor intensity is assigned to the respective MS^1^ precursor.

With PFΔ*Screen*, a single sample with a corresponding (optional) blank can be processed at a time. Blank correction is performed by setting an m/z and RT tolerance as well as a fold change with the desired increase of abundance in the sample compared to the blank. Features appearing in both sample and blank within the specified criteria are removed from the dataset. After preprocessing, the raw data is ready for specific PFAS prioritization. If feature finding by an external software is desired (e.g., vendor software), the following steps can also be performed by loading a feature table (.csv, that requires m/z, RT, and intensities of the [M] and [M+1] isotopes) into PFΔ*Screen* without feature detection via OpenMS. However, the raw mzML files are still needed to assign MS^2^ data to the features in the feature table (see SI). Besides pyOpenMS, the mass spectrometric Python library Pyteomics is used for selected calculations [50, 51].

#### PFASPrioritization

The PFAS prioritization workflow is intended in an iterative manner: after feature detection, the MD/C-m/C plot should firstly be manually inspected to determine reasonable boundaries to remove most of the detected features (e.g.,  ~ 90%) that cannot be PFAS due to their MD/C-m/C locations (depending on the underlying question). After determination of these cutoffs, the PFAS feature prioritization can be executed again focused on a subset of features, which will strongly decrease false positives in KMD analysis, fragment matching, and suspect screening where the respective parameters can be adjusted accordingly without a strong increase of wrong assignments. Since the execution time of PFΔ*Screen* is usually below 1 min (e.g., for  ~ 4000 spectra per sample), input parameters can easily be varied to test their influence on the outcome. After execution, a folder is generated named after the sample file where important results are saved, including a summary in an Excel sheet which is formatted as a table that can be easily inspected, sorted, and subset for a faster overview of the results as well as an additional CSV file that includes the same data (Fig. S2). Important plots are saved in the interactive HTML format which can easily be opened in any browser, allowing zooming and data inspection with interactive tooltips (Fig. S3).

In the workflow to prioritize features according to their likelihood of being PFAS, several pieces of evidence are calculated individually for all detected features in the first place. For all MS^1^ features, the number of carbon atoms, MD, and both MD/C and m/C dimensions are determined. To detect homologues series (HS), the KMD (with a predefined repeating unit required; e.g., CF_2_) is calculated and corresponding features belonging to a certain HS are aligned by providing a unique HS number (parameters: mass tolerance, minimum number of homologues).

For all MS^2^ spectra, fragment mass differences are calculated comprehensively. Therefore, all fragment differences within each MS^2^ spectrum are calculated and matched against a predefined list of PFAS typical mass differences (e.g., ΔCF_2_, ΔC_2_F_4_, ΔHF, ΔC_10_H_3_F_17_, more details can be found in [31]). This allows an efficient detection of fragments indicative for PFAS without prior knowledge on their actual mass [23, 31]. Furthermore, a list of typical PFAS diagnostic fragments (DFs, approximately 900 fragments) from literature are automatically matched with all fragmentation spectra (which is easily extendable) [52, 53]. Both negative and positive fragments are considered depending on the measurement polarity which can be specified in the GUI. The most important parameter is the MS^2^ noise threshold, used to specify the lowest MS^2^ intensity to be considered for DF, and mass difference matching. It is important to select a suitable instrument-specific threshold as a too low input value may result in a high number of false-positive annotations. Besides a mass tolerance for fragment matching, a minimal number of positive DFs or mass differences can be specified to flag a MS^2^ spectrum as potential hit.

To enhance annotation in the MS^2^, fragments that have a defined mass difference to another already annotated fragment (accurate mass match and therefore also a chemical formula) are also annotated by subtraction or addition of the respective mass difference (e.g., ΔC_2_F_4_) to an annotated chemical formula (e.g., C_12_H_5_F_12_O_4_S + ΔC_2_F_4_). This allows the calculation of unknown chemical formulas for fragment masses that are not present in the list of DFs (see Fig. S6).

In the third step, suspect screening by accurate mass match (with mass tolerance) can be performed. We used a custom PFAS suspect list format (.csv) which currently includes PFAS from the NIST suspect list [54]. This list which includes compound name, SMILES, chemical formula, and exact mass can easily be modified or extended with data from other suspect lists (e.g., from NORMAN or the CompTox Dashboard). For suspect screening, three adducts can be chosen which are [M–H]^−^ for negative polarity, and both [M+H]^+^ and [M]^+^ for positive polarity (compounds such as betaines present in various AFFF formulations are often detected as M^+^ ions) [55].

#### RawDataVisualization

After feature finding or the complete workflow, the MS raw data can be directly visualized via the PFΔ*Screen* GUI (Fig. 2 and S4).

##### EIC extractor

Extracted ion chromatograms (EICs) can be generated by accurate m/z (e.g., from the Excel or CSV results file) and inspected in an external window. Several masses can be extracted together (comma separated) to investigate coelution or RT shifts. To verify the systematic RT shifts of detected HS, a repeating unit can be specified (e.g., CF_2_) and *n* EICs are extracted at once (Fig. 2 and S4), allowing fast checking for reasonable peak shapes and elution order of suspected masses.

##### MS^1^ extractor

To visualize single MS^1^ spectra, a certain RT of interest can be specified. Theoretical isotope patterns of chemical formulas from suspect hits can then be plotted on top of the experimental MS^1^ isotope pattern (Fig. 2 and S4).

##### MS^2^ extractor

MS^2^ spectra can also be directly accessed via the GUI by inputting the accurate m/z value. If DFs and fragment mass differences were detected, they are displayed within the respective MS^2^ spectrum (Fig. S4).

##### EIC correlator

To detect potential in-source fragments (e.g., [M-HF]^−^) or adducts (e.g., [M+Br]^−^ or [M+Acetate]^−^) by coelution correlation, an m/z of interest can be specified and all detected features within a certain RT range are correlated (EICs) and only highly correlating ions can be visualized (e.g., correlation of *R*^2^ > 0.95). This can greatly enhance understanding of ionization processes and helps to find related ions that were not grouped during feature detection (more detailed explanation in the “Results and discussion” section, Fig. 6 and S9).

### Soil collection and extraction

To present the feature prioritization procedure via PFΔ*Screen*, four different PFAS-contaminated composite agricultural topsoil samples from Rastatt (R1 and R2) and Mannheim (M1, M2) regions (Germany) were extracted and measured by HPLC-QTOF-MS (see sampling details and soil physicochemical properties in the SI (S3)). The R1, R2, S1, and S2 soil names correspond to soils B, A, D, and H from Röhler et al. [56], respectively. Agricultural fields in these regions were subjected to contaminated paper sludge in the past and found to be highly contaminated with several PFAS classes [27, 32, 56]. Information on all chemicals used can be found in SI (S4). Soil extraction was adapted from existing procedures [27]. Briefly, 5 g of dried soil (40 °C) was weighed in 50-mL polypropylene (PP) tubes and combined with 10 mL of methanol (MeOH). The suspension was sonicated for 1 h and overhead shaken for 16 h. After centrifugation (10 min @ 4000 rcf), the supernatant was transferred into a 20-mL glass vessel, and extraction was repeated. The combined extracts (20 mL) were evaporated under a gentle stream of N_2_ until dryness at 40 °C and reconstituted in 1 mL of MeOH, sonicated for 10 min, and thoroughly vortexed for 1 min. In the last step, the enriched extract was filtered through a 0.2-µm regenerated cellulose syringe filter, transferred into PP HPLC vials, and stored in the fridge (4°C) until analysis. As quality control, an extraction blank following the identical extraction procedure but without adding any soil was prepared to account for background contamination.

### LC-HRMS measurements and data acquisition

Soil extracts were analyzed with an Agilent 1260 Infinity HPLC system (Poroshell 120 EC-C_18_ column; 2.1 mm × 100 mm; 2.7 µm particles at 40 °C) at a flow rate of 0.3 mL/min coupled to an Agilent 6550 QTOF-mass spectrometer. For compound separation, a 23-min gradient program was used (A: 95/5 H_2_O/MeOH + 2 mM NH_4_Ac; B: 5/95 H_2_O/MeOH + 2 mM NH_4_Ac) and both negative and positive measurements were performed (details in Table S1–S2). Data acquisition was performed in the data-dependent mode (ddMS^2^) using 3 scans/s (MS^1^ range: m/z 100–1700 and MS^2^ range m/z 70–1700) with a static exclusion list (resulting from prior MeOH blank injections) to avoid fragmentation of background signals. Furthermore, a rolling exclusion list was used to iteratively exclude previously triggered precursor masses from previous measurements (three injections) of the same sample to maximize the MS^2^ coverage. The threshold for precursor selection was set to 1000 counts, and each precursor was excluded for 0.5 min after collection of three MS^2^ spectra. For collision-induced dissociation, a linear m/z-dependent collision energy (CE) according to the following equation was used: $\mathrm{CE(}\mathrm{m/z) = 3}\frac{\mathrm{m/z}}{100}+ \mathrm{15 eV}$. To prevent sample cross contamination, a threefold needle wash in MeOH was performed in-between each injection. Each measurement sequence included several blanks and quality controls (PFAS reference standard mixture) to monitor instrument drift.

## Results and discussion

PFAS prioritization and identification with PFΔ*Screen* are aimed to be performed in an iterative process. This means that the program is executed multiple times allowing parameter adjustment to generate reasonable results. PFΔ*Screen* runtimes are usually below 1 min (e.g., for  ~ 4000 spectra per sample) for the whole workflow. When changing specific input parameters (e.g., tolerances, thresholds, mass differences), their effect on the output can directly be observed. In this way, input parameters can be conveniently adjusted depending on end-user needs and sample types. After feature detection, blank correction, and a short inspection of the results, the data can be reduced by the MD/C-m/C approach by setting an appropriate m/C cutoff value. Subsequent KMD analysis, fragment mass differences, DF matching, and suspect screening then result in a detailed table of a manageable size.

To demonstrate the PFΔ*Screen* workflow, it was applied here to four contaminated agricultural topsoils. The iterative identification process started with the soil extract of M1. After data preprocessing and application of prioritization techniques, the identified PFAS (including adducts and in-source fragments) were manually added to the suspect list, and the same workflow was applied to the next soil sample. In the following, the whole workflow starting from data reduction to final identification is discussed in detail.

### Data preprocessing

After data-dependent acquisition (DDA), the raw MS data (.d files, Agilent) were converted into mzML with MSConvert [40]. For each soil, PFΔ*Screen* was executed individually together with the extraction blank to remove background signals originating from both the extraction procedure and the HPLC system. The mass error for feature detection was set to 10 ppm, the MS^1^ intensity threshold was set to 2000 counts and the metabolites (5% RMS) isotope model from OpenMS was used to exclude features with unusual peak shapes of isotopic traces. Peaks reported after feature detection have to have a full width at half maximum (FWHM) above 1 s and below 1 min, and at least two isotopic traces. MS^2^ spectra were aligned with a mass tolerance of 5 mDa and an RT tolerance of 0.2 min (these tolerances can be verified by an interactive m/z vs. RT plot (Fig S3a)). Features detected in both sample and extraction blank that deviated by < 2 mDa at a RT difference of  < 0.1 min and were not at least fivefold more abundant in the sample were removed. Exemplified on soil M1, 4209 features were detected, which were reduced to 3750 features after blank correction in the ESI^−^ mode. A total of 1026 out of 2450 acquired MS^2^ spectra corresponded to detected features, from which 417 unique spectra remained (~ 11% MS^2^ coverage in first iteration).

### Data reduction by m/C and MD/C

**Fig. 3:**
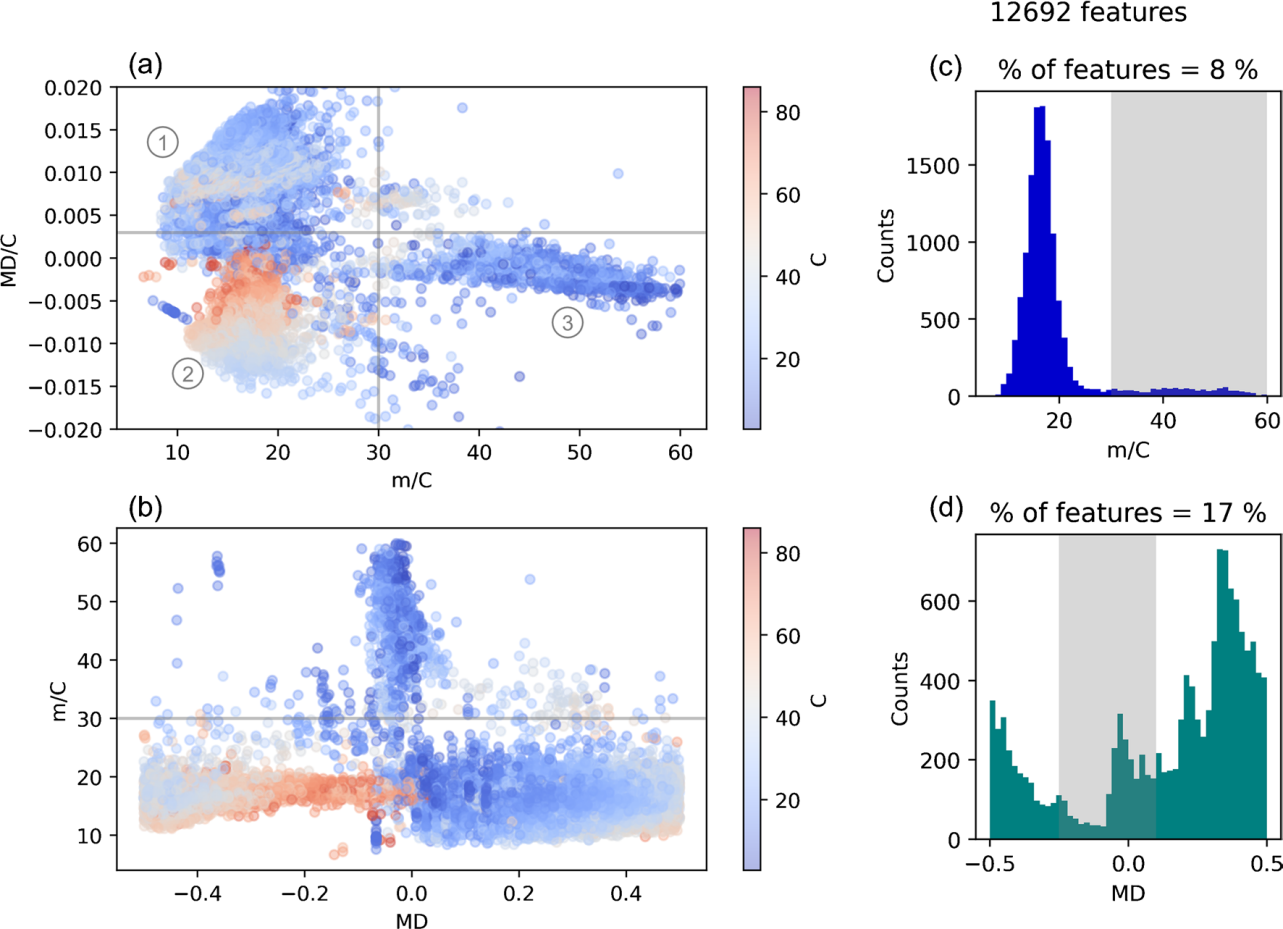
Data reduction by the MD/C-m/C approach compared to the MD. **a** MD/C-m/C plot for all detected features (12,692) in the four soil extracts and **b** m/C vs. MD. The colorbars correspond to the calculated carbon number. In the MD/C-m/C plot, potential PFAS (3) are clearly separated from hydrocarbons (1) and hydrocarbons with many carbon atoms that exceed a MD of  + 0.5 and are therefore flipped to a similar MD region as the PFAS but are easily separated by m/C. The number of features is reduced to 8% by the m/C dimension when cutting at m/C > 30 and to 7.4% when including a threshold of MD/C < 0.003 (gray lines in subplot a). **c** Histogram of m/C and of the MD (**d**), showing that the m/C works more efficiently than the MD (17% of the features remain when cutting at  − 0.25 < MD > 0.1 which includes 92% of the PFASOECDNA list [27]). Many features strongly exceeding a MD of  + 0.5 would be wrongly prioritized. Note how the m/C dimension allows a much clearer cutoff from hydrocarbon-based features compared to the MD

After these feature preprocessing steps, the m/C and MD/C dimensions were used for data reduction. When looking at the MD/C-m/C plot of all soils together (containing more than 12,000 features), a clear separation of three groups of compounds can be observed (Fig. 3a). Most features were located below m/C 30, which are a wide variety of different hydrocarbon molecules. A theoretical molecule exclusively consisting of (CH_2_)_n_ groups would be located at m/C = 14, while for the four soil extracts a clear peak distribution ranging from m/C ≈ 10–25 and reaching a maximum around m/C ≈ 16 was observed (Fig. 3c). The determination of the carbon number strongly depends on the peak picking algorithm, since it is based on robustly integrated EICs from the monoisotopic mass and its corresponding M+1 isotope (C ≈ I_M+1_/I_M_/0.011145). Therefore, a certain uncertainty should always be expected, which increases with decreasing ion abundance. Nonetheless, ~ 92% of all detected features are clearly located below m/C = 30 (e.g., humic substances) (Fig. 3c). Therefore, here a cutoff at m/C = 30 was chosen since PFAS that are dominated by fluorine usually have a higher m/C (e.g., m/C_6:2 diPAP_ ≈ 49; m/C_PFOA_ ≈ 51; m/C_6:2 FTAB_ ≈ 38). 6:2 FTAB is an AFFF constituent which already has a considerable fraction of hydrogen (C_15_H_19_F_13_N_2_O_4_S) compared to other PFAS, while other organic compounds containing less fluorine (compared to hydrogen, high H/F ratio) such as the pharmaceutical fluoxetine with only three fluorine atoms (C_17_H_18_F_3_NO, m/C ≈ 18) fall below the applied cutoff. Depending on the underlying NTS question, this cutoff can be adjusted accordingly. Attempting to remove further features, an MD/C cutoff of  <  + 0.003 was set, although as seen in Fig. 3a the m/C dimension was much more effective for data reduction. The MD/C-m/C approach was more efficient to reduce features compared to the MD, as shown in Fig. 3b and d. When applying a MD range from  − 0.25 to  + 0.1 Da, which would include 92% of the PFAS in the PFASOECDNA list (CompTox Dashboard [36, 57]), 17% of the features remained, while the combined m/C and MD/C cutoffs led to only 7.4% of remaining features. It is very important to note here that the number of features that strongly exceed a MD of  + 0.5 is not negligible, since a conventional calculation of the MD would result in a negative MD (e.g.,  − 0.2 Da for a saturated hydrocarbon with 60 carbon atoms (H(CH_2_)_60_H), whereas the true MD would be + 0.8 Da). As can be seen from the carbon number, a considerable number of features has more than 60 carbon atoms (up to 80 carbons) which are in a PFAS typical MD range (Fig. 3b). Therefore, setting an appropriate m/C cutoff is highly recommended, since these features are easily removed by this additional criterion. Eventually, when combining both m/C and MD/C cutoffs, only 949 features (7.4%) remain in all four soils together. This is an appropriate number of features for further PFAS-specific calculations such as KMD analysis, DFs, fragment mass differences, and suspect screening. It should be noted in particular that due to the removal of  ~ 90% of the initial features, the false-positive rate decreases drastically (especially with large lists) and allows adjustment of selected tolerances with smaller effect on false positives.

### KMD analysis, fragment differences, DFs, and suspect screening

**Fig. 4:**
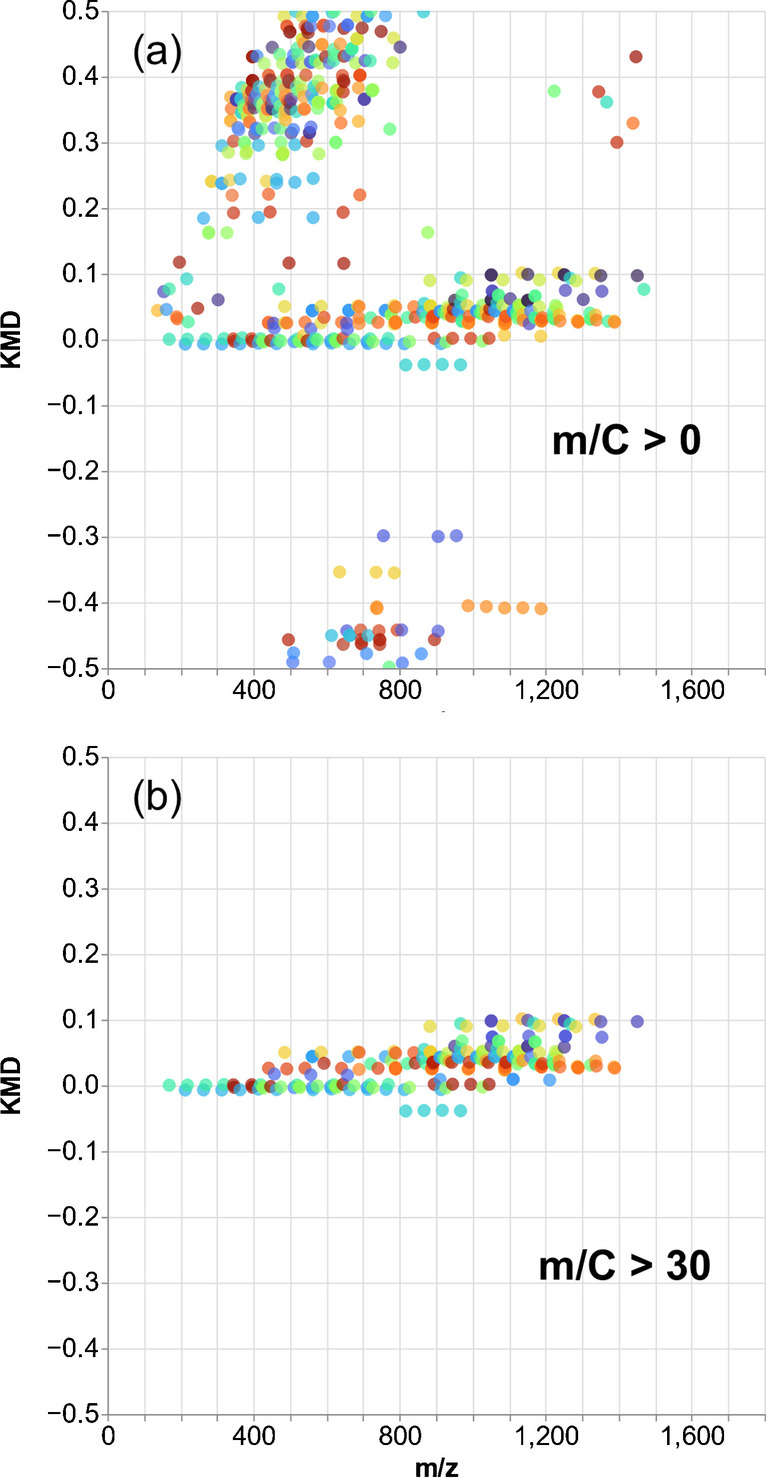
True- and false-positive CF_2_-based HS in soil M1 **a** without (m/C > 0) and **b** with m/C cutoff (m/C > 30). An MS^1^ noise threshold of 1000 counts was used for feature detection, and the KMD mass tolerance was set to  ± 1 mDa with a minimum of three homologues. Even with the low mass tolerance of  ± 1 mDa, many hydrocarbon matrix components are mimicking the CF_2_-repeating unit (see also Fig S5). Note: Multiple (CF_2_)_n_ differences within the KMD tolerance are also assigned to the respective HS; therefore, each datapoint has at least two HS partners

For further prioritization and tentative identification, repeating units representative for PFAS such as (CF_2_)_n_ and CF_2_O were applied to detect HS (mass tolerance was set at  ± 2 mDa, with at least 3 homologues). Without any m/C cutoff, in soil M1, 74 (CF_2_)_n_-based HS were detected, likely including numerous false positives (Fig. 4a) evidenced by a random RT pattern (no RT shift in linked KMD m/z vs. RT plot). The KMD analysis in PFΔ*Screen* is performed without checking the systematic RT shift, but the interactive KMD plot (HTML) allows a fast verification of RT shifts. Each HS can be highlighted individually by clicking on it, and the respective m/z vs. RT correlation is visualized (Fig. S5). Obviously, many hydrocarbon features were detected in the soil extract that are mimicking CF_2_-repeating units, which is a common issue of complex matrices [31, 37]. These compounds have a higher CF_2_-based KMD (e.g., 0.2 to 0.5, or lower if their MD strongly exceeds + 0.5 Da) compared to that of PFAS (Fig. 4a). If the combined MD/C-m/C cutoff is applied, the number of detected HS in soil M1 is reduced to 26 (~ 65% data reduction, see Fig. 4b) which confirms the utility of this approach.

For detection of fragment mass differences and DFs in the MS^2^ data, preliminary ΔCF_2_, ΔC_2_F_4_, ΔHF, and the list of DFs were used (later specific mass differences were searched). This resulted in the detection of 30 MS^2^ spectra that contained the specified mass differences, and 47 spectra with DF hits out of a total number of 373 unique MS^2^ spectra at a mass tolerance set to ± 2 mDa and an MS^2^ intensity threshold of 2000 counts in the M1 soil extract (first iteration).

In the suspect screening process, the hits by accurate mass (tolerance of 4 mDa) were reduced from 217 to 176 by the MD/C-m/C cutoff in soil M1.

### Manual identification process with the PFΔ*Screen* results table

The verification and (partially manual) identification process of prioritized features from the PFΔ*Screen* results table (Excel or CSV) was performed by sorting the table according to decreasing intensity, after removing features based on defined MD/C-m/C cutoffs. For soil M1, this resulted in a feature list with 305 potential compounds. Note that some features appear multiple times in the list due to structural isomerism, resulting in multiple features at multiple distinct RTs depending on the degree of separation and the peak finding algorithm. Each feature was verified manually for occurrence in the extraction blank and reasonable peak shape (until < 1% of the most abundant feature). Although a blank correction was performed, typical contaminations from the LC system with long tailing peaks can be integrated multiple times at different RTs. Therefore, they are not always correctly removed depending on the specified parameters. By using the RawDataVisualization tool of PFΔ*Screen*, EICs of every m/z belonging to one HS (using the integrated HS extrapolator) can be verified for RT shift and peak shape, eventually resulting in identification of homologues with very low abundances that were missed in the feature finding process due to the MS^1^ intensity threshold. The chemical formulas from suspect hits were used to check for reasonable isotope patterns with the RawDataVisualization of PFΔ*Screen.* SMILES codes were used to verify at least one candidate per HS by an MS^2^ spectrum.

**Fig. 5:**
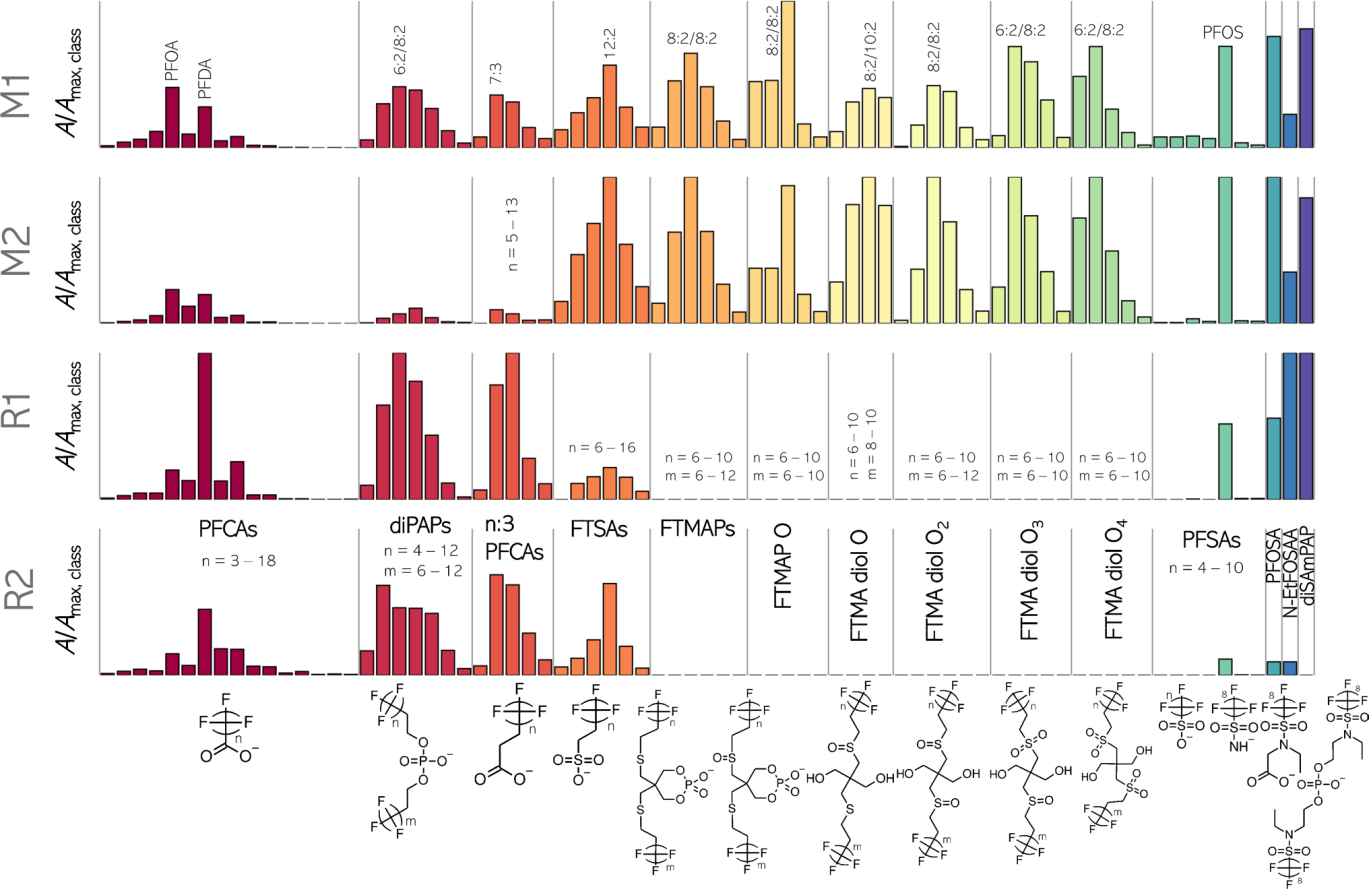
Qualitative summary of identified PFAS in the four soils (M1, M2, R1, and R2). Each class (e.g., PFCAs, diPAPs) is normalized to the peak area of the most abundant homologue within all four samples. Further abbreviations: FTMAP O, FTMAP-sulfoxide; FTMA diol O, FTMA-diol-sulfoxide; FTMA diol O_2_, FTMA-diol-disulfoxide or -sulfone; FTMA diol O_3_, FTMA-diol-sulfoxide-sulfone; FTMA diol O_4_, FTMA-diol-disulfone. Note that depending on chain length and sulfur oxidation degree, diPAPs, FTMAPs, and FTMA-diols were detected as complex mixture of structural and positional isomers (e.g., 6:2/10:2 ≠ 8:2/8:2, or disulfoxide ≠ sulfone). Very small abundant identifications and triPAPs are not shown in the figure

In total, nine PFAS classes could be identified via PFΔ*Screen* in the four soils that exhibited at least one suspect hit per HS or compound (Fig. 5). Perfluoroalkyl carboxylic acids (PFCAs, C_4_–C_20_), fluorotelomer alkyl phosphate diesters (diPAPs, 4:2/6:2–12:2/12:2), n:3 fluorotelomer carboxylic acids (FTCAs, 5:3–13:3), fluorotelomer sulfonic acids (FTSAs, 6:2–16:2), perfluorosulfonic acids (PFSAs, C_4_–C_10_), perfluorooctane sulfonamide (PFOSA), N-ethylperfluoro-1-octanesulfonamidoacetic acid (N-EtFOSAA), and N-ethyl perfluorooctane sulfonamide ethanol–based phosphate diester (diSAmPAP) were identified in all four soils. Different chain length distributions and abundances were observed (Fig. 5). diPAPs were detected as complex mixtures of several structural isomers depending on their chain length (e.g., 6:2/10:2 and 8:2/8:2, shown by MS/MS). Additionally, their EICs showed peaks at much later RTs corresponding to in-source fragments of triPAPs (Fig. S7). While all telomer-based PFAS were detected as linear chains, the PASF-based PFAS (PFSAs, N-EtFOSAA, PFOSA, and diSAmPAP) showed typical chromatographic peak shapes of mixtures of branched and linear isomers [58]. In these cases, the dominance of a C_8_-based chemistry can be observed (see PFSAs in Fig. 5).

All four soils had a similar contamination pattern. However, for soils M1 and M2 (Mannheim region), another very abundant precursor class, namely FTMAPs, was detected (including isomeric profiles ranging 6:2/6:2 to 10:2/12:2), as well the previously identified TPs FTMAP-sulfoxides [31].

The 6:2 fluorotelomer mercapto alkyl phosphate esters (6:2/6:2 FTMAP) could be confirmed with an in-house synthesized reference standard, leading to identification levels of 1 for 6:2 FTMAP and 2a for the further homologues due to clear MS/MS evidence [59]. In general, all identified PFAS are in good agreement with previous studies including biotransformation that characterized other soil samples from both Rastatt and Mannheim [27, 31, 32, 39, 56].

The PFΔ*Screen* results table also revealed several unknown HS that were detected but did not have an accurate mass match with the suspect list. Their identification with the help of the EIC correlator of PFΔ*Screen* is discussed in the following.

### EIC correlator: coelution correlation analysis for identification of unknowns

**Fig. 6:**
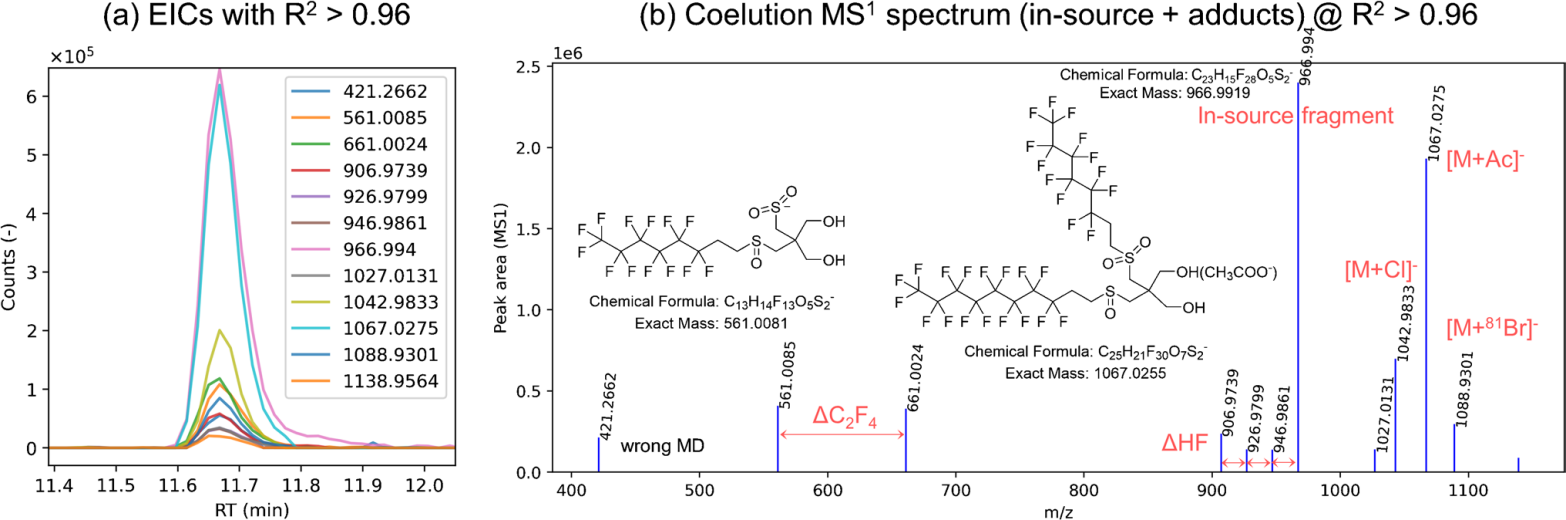
Detection of coeluting in-source fragments and adducts via the EIC correlator of PFΔ*Screen* for the identification of 6:2/8:2 FTMA diol sulfoxide sulfone. The EIC of the unknown in-source fragment m/z = 966.9944 (which was detected as one member of a HS via KMD) was correlated with all EICs eluting at its RT ± 25 s resulting in non-targeted detection of related ions. In total, 4 HS corresponding to 21 novel FTMAP TPs were identified via the use of this tool (see Fig. 5). A RT shift with increasing oxidation degree (1 O up to 4 O) was observed due to increasing polarity. Note that the EICs of [M+^37^Cl] and [M+^79^Cl] are also in the raw MS^1^ spectra; however, they were combined into one feature by feature finding algorithm of pyOpenMS (in case of Br, a wrong isotope grouping occurred) and therefore not detectable by the correlation analysis

After identification of the PFA*Screen* results, there were several C_2_F_4_-based HS left without any hit in the suspect list. When looking at several MS^1^ spectra of different homologues, many coeluting ions were observed, often characterized by HF losses and other mass differences (Fig. S8). This is an indication of in-source fragmentation of these classes [60, 61]. To be able to efficiently group corresponding in-source fragments and potential adduct ions together, the EIC correlator from the raw data visualization tools of PFΔ*Screen* was used to correlate the EICs of suspected features (from a given HS) with the EICs of all detected features that coelute within a given RT range of  ± 25 s. Strong correlation of EICs can be used to detect related ions and allows their isolation from other ions in consecutive MS^1^ spectra without knowing their mass differences [62–65]. This is exemplified on the unknown m/z 966.9944 which is a member of a suspected HS. When correlating the EIC of m/z = 966.9944 with the EICs of all coeluting features within a RT range of 50 s, 12 out of 368 EICs correlated with an *R*^2^ > 0.96 at an extraction width of 5 mDa (see Fig S9 for more details). The result is an MS^1^ spectrum that only contains coeluting ions (correlation spectrum) of several in-source fragments and adducts (Fig. 6). Since well-known mass differences such as ΔC_2_F_4_ and ΔHF were found in this MS^1^ spectrum, a telomer-based PFAS with potentially two telomer chains (e.g., 6:2/8:2) was suspected [31]. When looking at the mass differences of detected coeluting ions, [M+Cl]^−^, [M+Br]^−^, and [M+Ac]^−^ adducts and several other in-source fragments could be observed. The detection of [M+Cl]^−^ and [M+Br]^−^ ions was of great importance since they allowed the determination of [M] rather easily which then also allowed the identification of other adducts and the molecular formula. The m/z = 966.9944 (in-source fragment) corresponds to a FTMAP-related substance, which was tagged FTMA-diol-sulfone-sulfoxide or FTMA-diol-O_3_ (see Figs. 5 and 6). With this correlation technique, several tens of unknown HS could be grouped into four novel FTMAP-related compound classes (Fig. 6). They were identified with one oxygen (sulfoxide) and up to 4 oxygens (disulfone) and to the best of our knowledge not reported in literature before. They could be microbial or photochemical FTMAP TPs and close the unknown gap in a previous FTMAP-related transformation study [66], or they could be used intentionally or as side-products in PFAS-coated papers that contaminate these soils. These kinds of correlation spectra made identification possible since the MS^2^ spectra of the adducts ([M-H]^−^ ions of the FTMA-diols were not detected at all which makes sense with ESI) barely formed useful fragments except for Br^−^ which made them hard to interpret. The use of in-source fragments for identification has the advantage that isotope patterns are available for all ions (features), which is often not the case in MS^2^ spectra depending on the isolation width of the precursor ion. All these FTMAP-related substances form multiple in-source fragments (and adducts), all could be confirmed with rather high confidence (identification level of 2b). They all could be grouped by C_2_F_4_- and O-based KMD (for O-KMD, see Fig. S10) with systematic RTshifts, besides eluting at higher RT than FTMAPs due to their lower polarity attributed to the loss of the phosphoric acid group.

## Conclusions

PFΔ*Screen* can be used efficiently for prioritizing features in both LC- and GC-HRMS (ESI and APCI) raw data in all kinds of samples independent of the vendor of the mass spectrometer used. Especially, the MD/C-m/C approach is a powerful tool to drastically decrease the number of features and thus reduce false-positive assignments, overcoming a common issue during NTS. Due to the short computational time of PFΔ*Screen* (less than 1 min for 4000 spectra), input parameters can be conveniently adjusted depending on the tested sample, instrument used, and end-user needs. Since the number of unknown PFAS in complex environmental and technical samples is still unknown, NTS approaches that combine several data reduction techniques for an efficient workflow are of importance to comprehensively elucidate the identity occurrence and fate of organic pollutants such as PFAS.

### Supplementary Information

Below is the link to the electronic supplementary material.

Supplementary file1 (PDF 2.59 MB)

## Data Availability

The Python source code of PFΔ*Screen* is available on GitHub (https://github.com/JonZwe/PFAScreen) together with example files. It is published under the LGPL-2.1 license.
