# Supplementary material for: PFΔScreen — an open-source tool for automated PFAS feature prioritization in non-target HRMS data
Source: Anal Bioanal Chem. 2023 Nov 30;416(2):349–62. doi: 10.1007/s00216-023-05070-2 (PMC10761406; doi:10.1007/s00216-023-05070-2)
Supplement: Supplementary file 1 — Supplementary file1 (PDF 2.59 MB) [file 216_2023_5070_MOESM1_ESM.pdf]

Supporting Information

PFΔ*Screen* – An open-source tool for automated PFAS feature prioritization in non-target HRMS data

Jonathan Zweigle,[+,*] Boris Bugsel,[+] Joel Fabregat-Palau,[‖] Christian Zwiener[+,*]

[+]Environmental Analytical Chemistry, Department of Geosciences, University of Tübingen, Schnarrenbergstraße 94-96, 72076 Tübingen, Germany

[‖]Hydrogeochemistry, Department of Geosciences, University of Tübingen, Schnarrenbergstraße 94-96, 72076 Tübingen, Germany

[*]*Corresponding authors*

Contents

S1 INSTALLATION OF PFΔSCREEN 2
S2 PFΔ*SCREEN* FUNCTIONALITY AND OUTPUT 3
Fig. S 1: Current graphical user interface (GUI) of PFΔ*Screen* which is separated into three main functionalities. 3
Fig. S 2: PFΔ*Screen* results table (here as formatted Excel table, a CSV file is also provided). 5
Fig. S 3: PFΔ*Screen* interactive HTML plots. 5
Fig. S 4: PFΔ*Screen* interactive figure from the RawDataVisualization tool. 6
Fig. S 5: Interactive KMD tooltips to visualize RT-shifts with increasing m/z for each detected HS. (a) Systematic (fits to PFCAs) and (b) non-systematic RT-shift (potential false-positive unknown group of compounds). 7
Fig. S 6: Example of an MS^2 spectrum where unknown chemical formulas (here only C_8F_{17}) of fragments are calculated by propagation of chemical formulas from diagnostic fragments via fragment mass differences. 7
Figure S 7: EIC of 6:2/6:2 diPAP (RT = 9.6) with in-source fragments that correspond to isomeric triPAPs (e.g., 6:2/6:2/6:2 triPAP). 8
Fig. S 8: HF mass differences from in-source fragmentation of FTMAP-related compounds (e.g., m/z = 1251.0202 which is an $[M+Acetate]^-$ ion of FTMAP diol disulfoxide/sulfone, $C_{27}H_{18}F_{38}O_4S_2(CH_3COO)^-$ = 1251.0172). 8
Fig. S 9: Results from the EIC correlator from the RawDataVisualization tools of PFΔ*Screen* for the in-source fragment m/z = 966.9944 8
Fig. S 10: Cutout from an O- and CF_2-based KMD vs. m/z plot from the soil extract of M1 showing the different sulfur oxidation states from one to four oxygen atoms. 9
S3 SOIL SAMPLING 9
S4 CHEMICALS 9
S5 INSTRUMENTAL PARAMETERS 10
Table S1: Gradient elution of the HPLC-QTOF method. 10
Table S2: Summary of instrument and scan source parameters used for HPLC-QTOF measurements. 10
REFERENCES 10

S1 Installation of PFΔScreen

PFΔ*Screen* can be installed and executed within the standard Python environment or by using the Anaconda distribution. To make installation and use as easy as possible, PFΔ*Screen* can be automatically installed with the `Installation.bat` file and executed with the `Run_PFAScreen.bat` file. Of course, people familiar with Python can execute the source code with their own custom environment and editor. In the following, the two steps needed for a simple installation are explained.

1) **Download PFΔ*Screen*:** Download the PFΔS*Screen* source code from https://github.com/JonZwe/PFAScreen by clicking on the green "Code" button and click "Download ZIP". When downloaded, unzip the folder and move it to a local folder on your computer.
2) **Automatic installation of Python and the required packages with Installation.bat:** Navigate into the folder where PFΔS*Screen* was copied (PFAScreen-main). Double click the `Installation.bat` file. Note that depending on your Windows safety settings a warning notification might open that needs to be accepted. The Windows command line interface will open, and the Microsoft Store opens automatically if you do not have Python installed on your computer. Click on "Install" and wait until the installation of Python is finished and close the Microsoft Store. Back to the Windows command line press any button to automatically install pip (Package Manager for Python) and in the following all required Python packages. Finally, when the message "Installation successfully finished" pops up, press any button and the installation is completed.

Note that the Python source code (without the automatic installation via batch files) can also executed on other operating systems within the Python environment. Here, the respective packages need to be installed manually.

S2 PFΔ*Screen* functionality and output

To start PFΔ*Screen*, double click the `Run_PFAScreen.bat` file. Note that depending on your Windows safety settings a warning notification might open that needs to be accepted. Both the GUI and a Python console window will open (Fig. **S1**). To load a MS raw datafile, click the "Browse Sample.mzML" button (see Nr. 1 in Fig **S1**) and choose the mzML file of a sample and an optional mzML file of a blank control (Browse Blank.mzML).

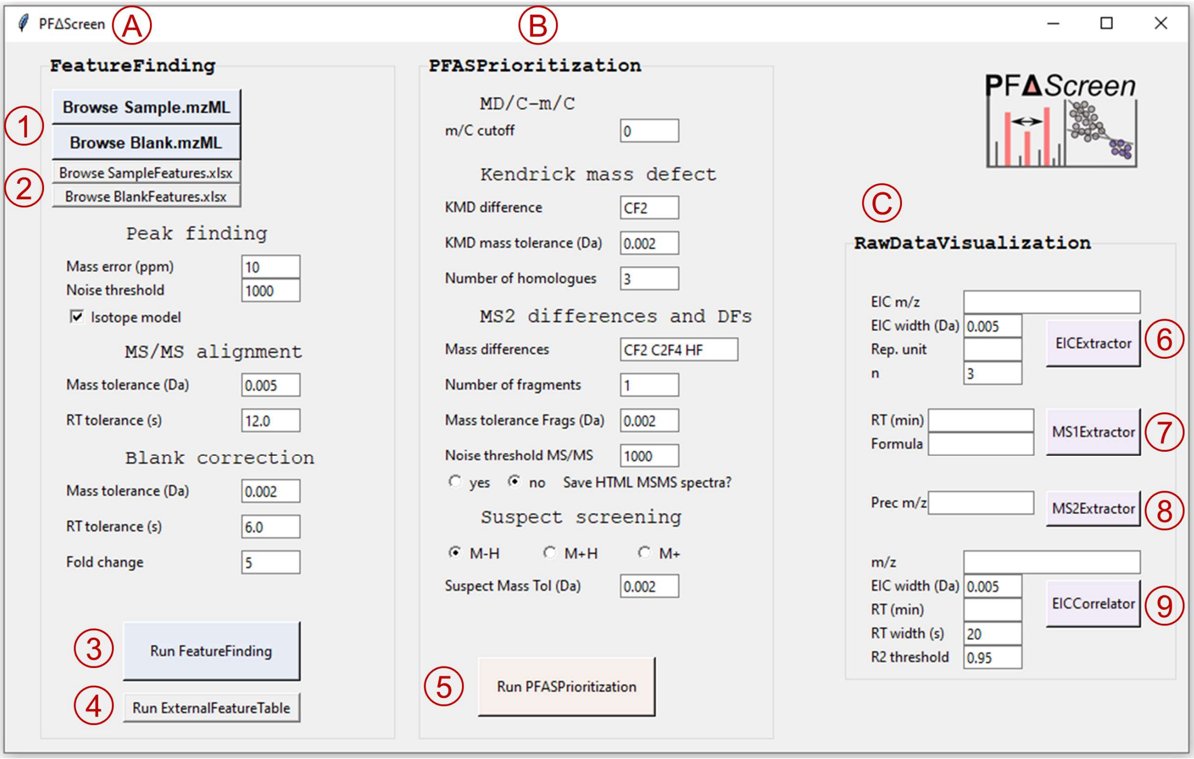

Fig. S 1: Current graphical user interface (GUI) of PFΔ*Screen* which is separated into three main functionalities. (A) FeatureFinding to detect features in MS raw data, align MS2 spectra to detected features and perform a basic blank correction (optional: reading a custom feature list (.csv) from feature finding by another software); (B) PFASPrioritization to prioritize the features according to several techniques such as the MD/C-m/C approach, KMD analysis, fragment mass differences, diagnostic fragments and suspect screening resulting in output of several plots and a summary table (Excel and CSV); (C) RawDataVisualization to visualize MS raw data (EICs, MS^1 and MS^2 spectra) and correlate related ions to detect in-source fragments and adducts.

The samples should have been measured under data-dependent acquisition ($ddMS^2$) with centroided spectra, ideally with one collision energy per precursor. Now, the parameters for peak finding, MS^2 alignment and blank correction can be specified and executed by pressing the "Run FeatureFinding" button (Nr. 3 in Fig **S1**). Since OpenMS and our MS^2 alignment and blank correction code are rather fast this task usually takes less than one minute (e.g., for

4000 spectra per sample), and runtime is mainly dependent on the selected MS^1 noise threshold. In case another feature finding procedure (e.g., from vendor software) is desired, custom feature lists (see `external_feature_list.xlsx` on GitHub) together with the respective mzML files can instead be included in PFΔ*Screen* (without peak finding by OpenMS). This is done by the "Browse SampleFeatures.xlsx" and "Browse BlankFeatures.xlsx" buttons, which are preprocessed by the "Run ExternalFeatureTable" button (Nr. 2 and 4 in Fig **S1**). Note that data evaluation only works when the corresponding mzML files are also given; otherwise MS^2 data would be missing. Whenever the **`FeatureFinding`** tab is completed, the **`RawDataVisualization`** (C in Fig **S1**) can be used even without PFAS-specific data. To perform the **`PFASPrioritization`** (B in Fig **S1**), appropriate input parameters can be set, and then PFAS-specific data evaluation is performed by clicking the "Run PFASPrioritization" button (Nr. 5 in Fig **S1**). This task is usually computed in less than one minute, allowing a convenient adjustment of input. Afterwards, MS^2 spectra displayed by the **`RawDataVisualization`** tool (MS^2 extractor), have highlighted fragment mass differences and diagnostic fragments, if some were detected. After executing the **`PFASPrioritization`** tab, the PFΔ*Screen* results table (Excel format and additional CSV file, Fig. **S2**) and several interactive HTML plots (Fig. **S3**) are saved in a folder named after the sample that can be easily inspected, including a MD/C-m/C plot, a m/z vs. RT plot (with and without MS^2 raw data), a KMD with linked m/z vs. RT plot (to verify systematic RT-shifts), and a m/C histogram. Data from the results table can be used to visualize EICs (and extrapolate HS with common repeating units such as CF_2), MS^1 and MS^2 spectra. Additionally, a coelution correlation can be performed with the **`RawDataVisualization`** tool. Also, the theoretical isotope patterns of suspect hits can be displayed over the experimental isotope patterns (MS^1) (see Fig. **S4**).

	A	B	C	D	E	F	G	H	I	J	K	L	M	N	O	P	Q	R
1	Index	m/z	m/z+1	m/z intens	RT (m	RT	n_diffs	n_dias	C	MD	MD/C	m/C	KMD	HS Number	Unique Hom	hit_in_list	FORMULA	SMILES
2	2734	498.93062	499.933368	2273744.487	7.29678	437.807	0	0	7.112	-0.069	-0.00976	70.149	-0.03752	5528	2	['Heptadecafl	['C8HF17O3S',	['C(C(C(C(
3	4909	900.9085	901.912121	112198.5211	7.65217	459.13			13.53	-0.091	-0.00676	66.569	-0.03395	51	2	['Bis(heptade	['C16HF34O2P	['C(C(C(C(
4	4030	669.96207	670.96497	1260132.474	9.81982	589.189	0	1	10.55	-0.038	-0.0036	63.529	0.004859	2180	1	['N-methylpe	['C13H6F21NO	['CN(CC(=
5	4294	714.95517	715.958482	2146242.525	9.81982	589.189	10	4	11.3	-0.045	-0.00397	63.276	0.000834	1584	1			
6	89	168.98971	169.993094	3074503.186	4.00965	240.579	0	0	2.732	-0.01	-0.00377	61.853	0.000505	2012	5	['PFBA-CO2H ,	[nan]	[nan]
7	3793	636.64176	637.644494	374695.8686	11.6856	701.134			10.35	-0.358	-0.0346	61.487	-0.31757	4084	1			
8	2405	466.97464	467.978318	341997.3838	6.977	418.62			7.595	-0.025	-0.00334	61.482	0.004469	1792	2			
9	2735	498.93101	499.934109	6416312.064	7.43892	446.335	10	8	8.121	-0.069	-0.0085	61.436	-0.03712	5528	2	['Heptadecafl	['C8HF17O3S',	['C(C(C(C(
10	4073	676.61431	677.617707	129699.9687	11.6856	701.134			11.03	-0.386	-0.03495	61.318	-0.34247	2952	1			
11	5	110.9759	111.978379	1708890.157	12.0765	724.587			1.822	-0.024	-0.01323	60.916	-0.01701	1152	1			
12	4703	816.90908	817.913829	89497.34175	7.36783	442.07			13.48	-0.091	-0.00674	60.587	-0.03874	1786	4			
13	2851	510.96415	511.967239	324169.5619	6.977	418.62			8.629	-0.036	-0.00415	59.218	-0.00321	1153	4	['Hydrogen-su	['C10H2F18O3'	['C(C(C(C(
14	4813	866.90665	867.910247	370298.3747	7.59885	455.931			14.67	-0.093	-0.00636	59.101	-0.03798	1786	4			
15	4893	894.94479	895.948189	719920.3489	15.7741	946.446			15.38	-0.055	-0.00359	58.175	0.001951	5016	14			
16	3817	640.63765	641.640766	93397.00261	11.4546	687.275			11.12	-0.362	-0.03257	57.591	-0.32143	4536	1			
17	4957	916.90365	917.906737	311725.1191	7.7943	467.658			15.97	-0.096	-0.00603	57.426	-0.03778	1786	4			
18	2726	497.94682	498.948762	342366.7266	8.29177	497.506			8.686	-0.053	-0.00612	57.327	-0.02137	5482	2	['Perfluorooc	['C8H2F17NO2	['C(C(C(C(

Fig. S 2: PFΔ*Screen* results table (here as formatted Excel table, a CSV file is also provided). This table summarizes most calculations performed in the PFAS feature prioritization steps and is directly formatted as a table to conveniently sort and slice data. m/z and RT values can easily be copied and for instance EICs or MS spectra (and coelution correlation) can be visualized in the RawDataVisualization tool of PFΔ*Screen.*

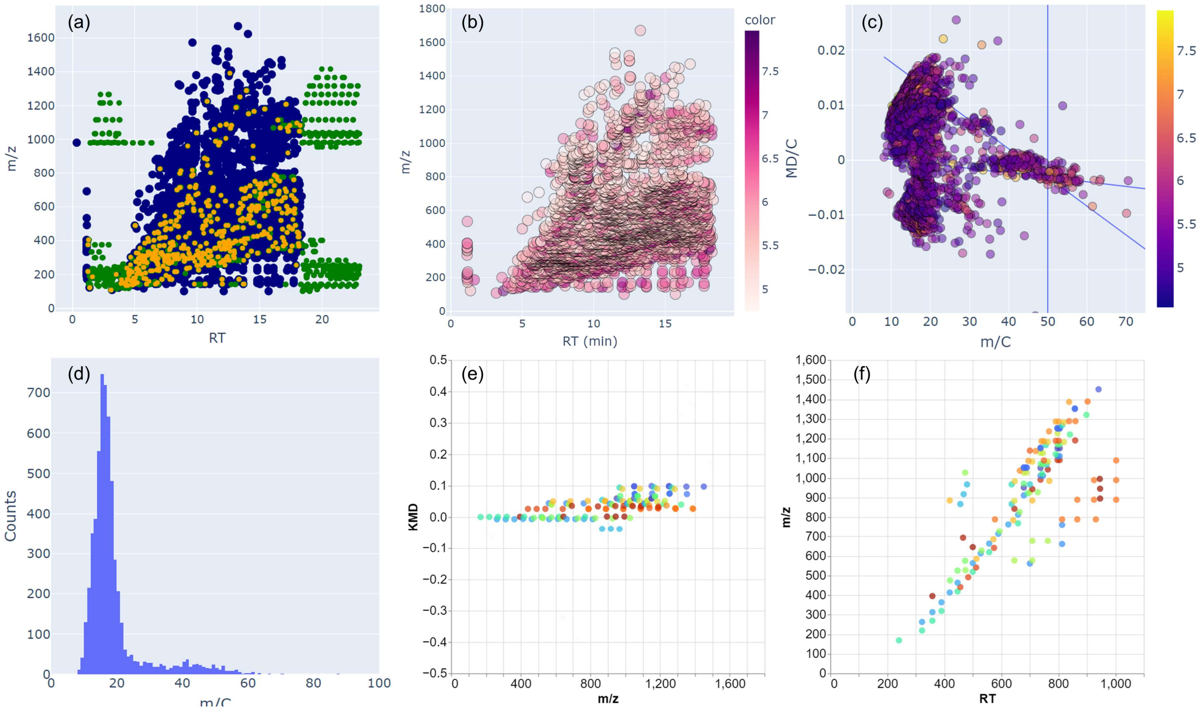

Fig. S 3: PFΔ*Screen* interactive HTML plots. (a) m/z vs. RT of all features and the precursors with MS2 raw spectra. Blue color corresponds to a detected feature, yellow if an MS^2 spectrum was assigned and green displays all MS^2 spectra. This plot can be used to find suitable m/z and RT tolerances for MS^2 alignment depending on the chromatography (e.g., peak width) and the MS^2 scan rate. (b) m/z vs. RT overview with m/C as colormap. (c) MD/C-m/C plot to deduce reasonable cutoffs for data reduction depending on the sample matrix. (d) m/C histogram to visualize the m/C distribution of the measured sample. (e) KMD plot coupled to (f) m/z vs. RT to easily verify the systematic RT-shift of each detected homologous series (see Fig. **S5**).

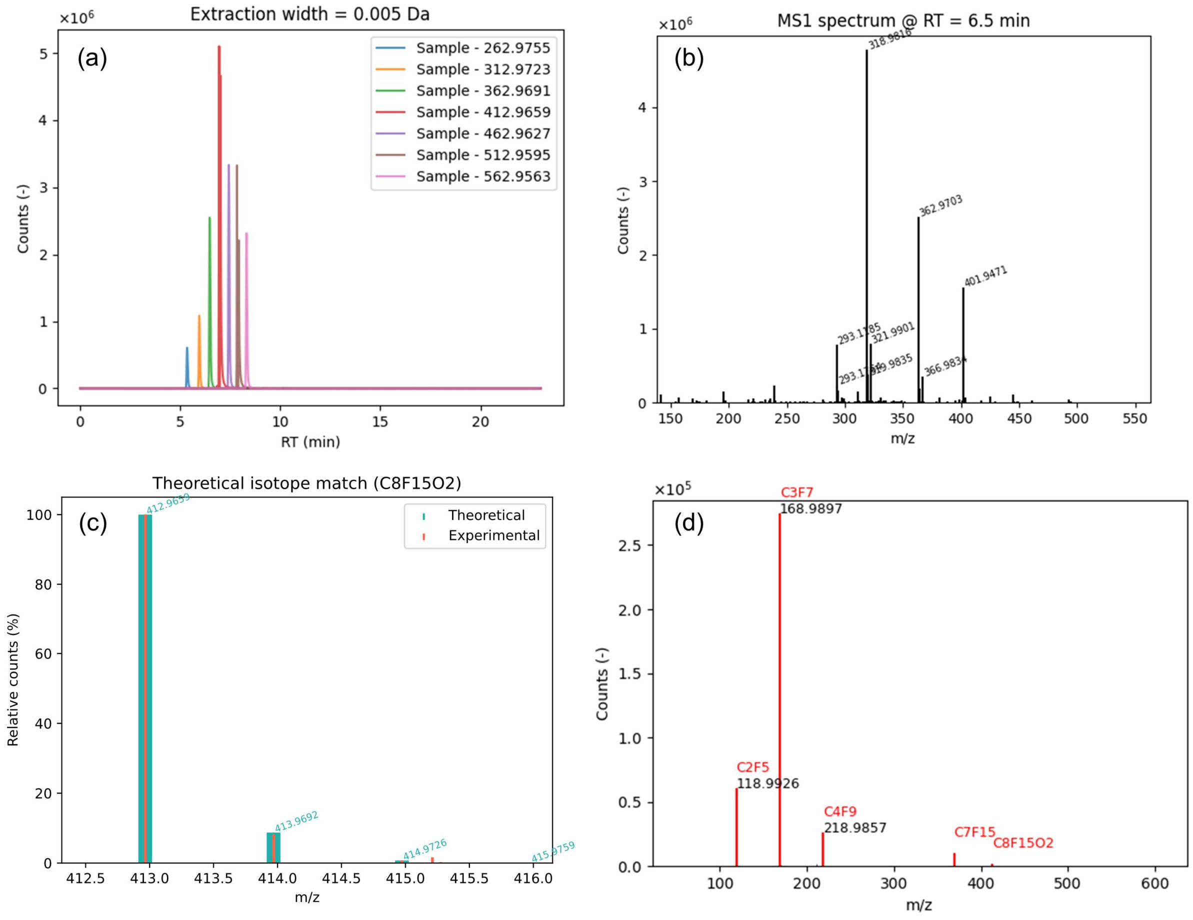

Fig. S 4: PFΔ*Screen* interactive figure from the RawDataVisualization tool. (a) EICs can be generated with comma separated lists of m/z values of for one m/z value *n* homologue of a common repeating unit (e.g., CF_2) are automatically generated. (b) Extracted MS^1 spectrum at a particular RT of interest. (c) When a chemical formula of a suspected compound (e.g., a suspect hit for PFOA, $C_8F_{15}O_2$ for $[M\text{-}H]^-$) is given, the theoretical isotope pattern is overlayed with the normalized cutout at this specific m/z. (d) MS^2 spectra of an m/z of interest can also be visualized with annotations and fragment mass differences.

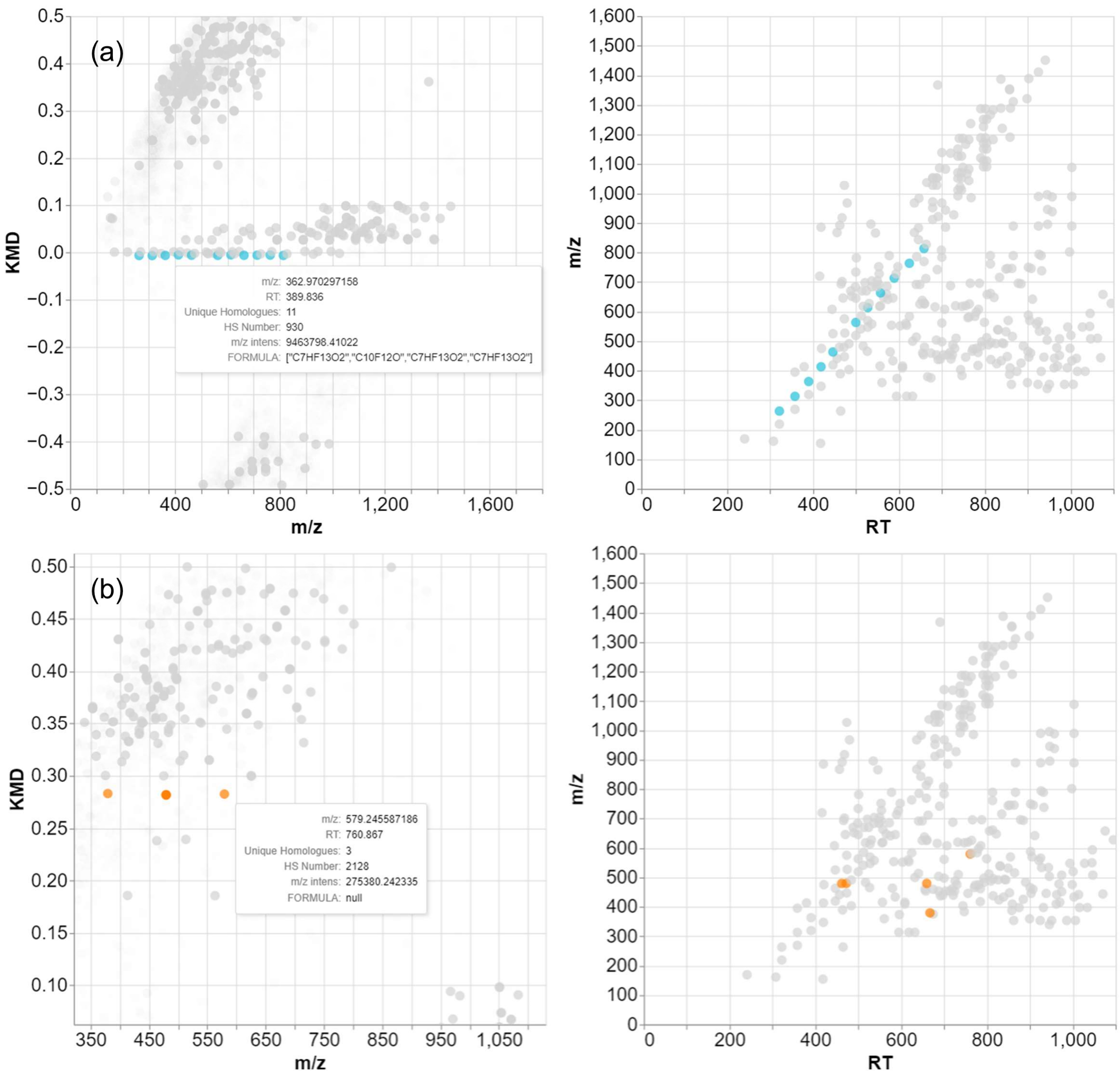

Fig. S 5: Interactive KMD tooltips to visualize RT-shifts with increasing m/z for each detected HS. (a) Systematic (fits to PFCAs) and (b) non-systematic RT-shift (potential false-positive unknown group of compounds).

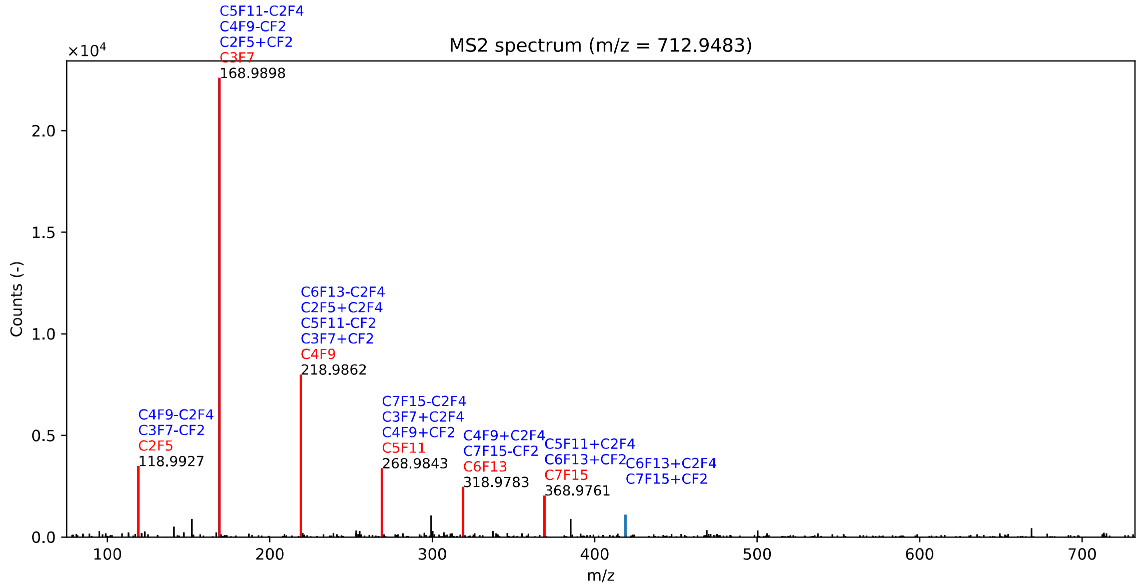

Fig. S 6: Example of an MS^2 spectrum where unknown chemical formulas (here only C_8F_{17}) of fragments are calculated by propagation of chemical formulas from diagnostic fragments via fragment mass differences.

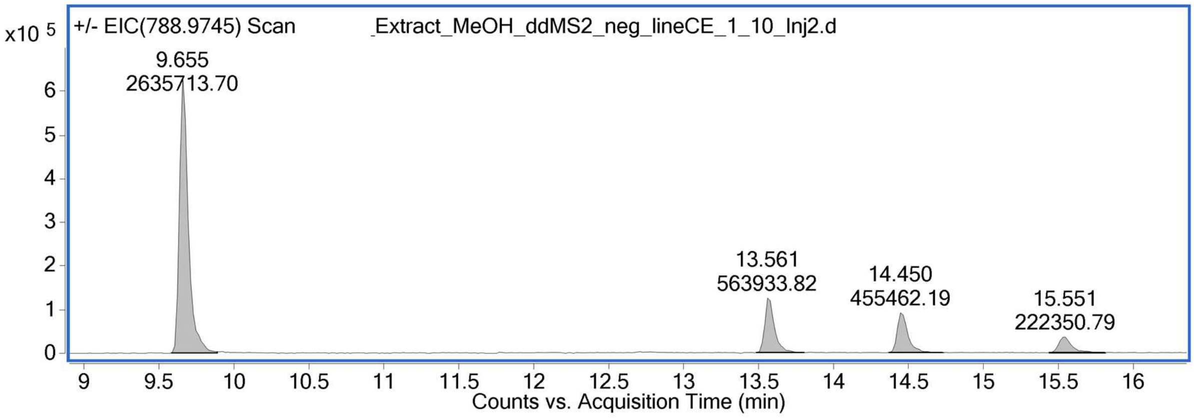

Figure S 7: EIC of 6:2/6:2 diPAP (RT = 9.6) with in-source fragments that correspond to isomeric triPAPs (e.g., 6:2/6:2/6:2 triPAP).

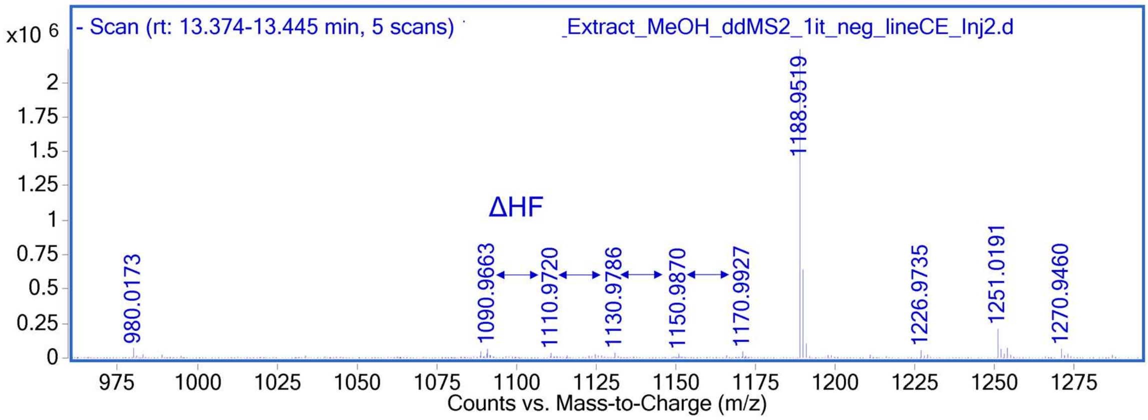

Fig. S 8: HF mass differences from in-source fragmentation of FTMAP-related compounds (e.g., m/z = 1251.0202 which is an [M+Acetate]$^-$ ion of FTMAP diol disulfoxide/sulfone, $C_{27}H_{18}F_{38}O_4S_2(CH_3COO)^-$ = 1251.0172).

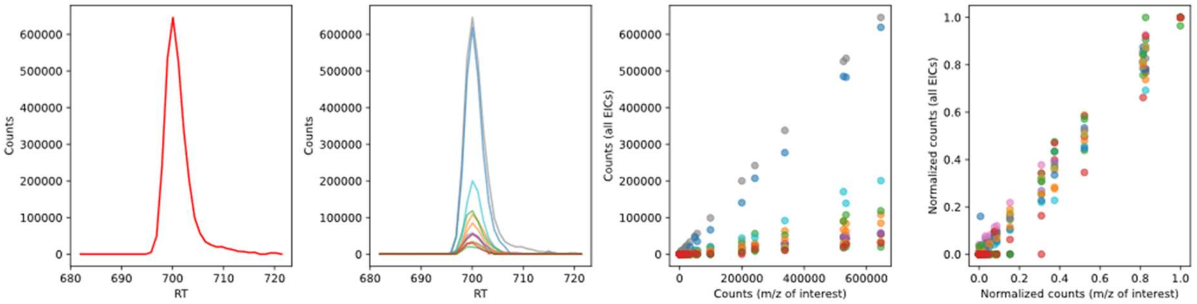

Fig. S 9: Results from the EIC correlator from the RawDataVisualization tools of PFΔ*Screen* for the in-source fragment m/z = 966.9944 (that corresponds to 6:2/8:2 FTMA diol sulfoxide sulfone) at a RT-width of 20 s and a R2 correlation threshold of > 0.95.

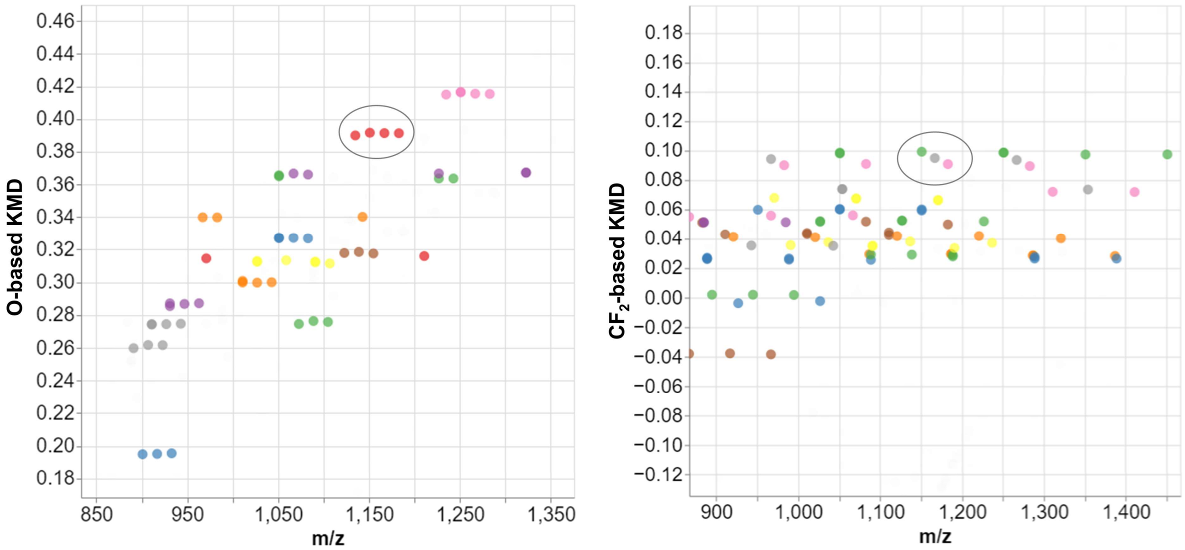

Fig. S 10: Cutout from an O- and CF_2-based KMD vs. m/z plot from the soil extract of M1 showing the different sulfur oxidation states from one to four oxygen atoms.

S3 Soil sampling

Soils were sampled on four agricultural fields in one diagonal over the respective area. Soils R1 and R2 were sampled near Rastatt, both within the 0 – 30 cm horizon. Soil R2 was a sandy loam soil, with pH 5.5 and organic content of 0.8%. Soil R1 was a loamy sand soil, with pH 6.9 and organic content of 2.3%. Soils M1 and M2 were sampled near Mannheim within the 0-30 and 0-50 cm horizon, respectively. Soil M1 was a loam soil, with pH 7.1 and organic content of 6.6%. Soil M2 was a clay loam soil, with pH 7.0 and organic content of 3.9%. All samples were homogenized and mixed thoroughly [1].

S4 Chemicals

Water and methanol (MeOH) were both LC-MS grade (Fisher Chemical). Ammonium acetate (NH_4Ac, ≥99%) was purchased from Fisherbrand. Reference standards of PFCAs, PFSAs, 5:3 FTCA, 6:2 and 8:2 FTSA, and 6:2 and 8:2 diPAP, PFOSA, N-EtFOSAA, and diSAmPAP were purchased from Wellington laboratories. 6:2 FTMAP was previously synthesized (details in [2]).

S5 Instrumental parameters

Table S1: Gradient elution of the HPLC-QTOF method. A = 95/5 H_2O/MeOH + 2 mM NH_4Ac and B = 95/5 MeOH/H_2O + 2 mM NH_4Ac.

Time (min)	A (%)	B (%)
0	85	15
2.0	30	70
5.0	10	90
10.0	0	100
15.0	0	100
15.1	85	15
22.0	85	15

Table S2: Summary of instrument and scan source parameters used for HPLC-QTOF measurements.

**Instrument Parameters**	
Gas Temp (°C)	150
Gas Flow (L/min)	16
Nebulizer pressure (psig)	35
Sheath gas temperature (°C)	380
Sheath gas flow (L/min)	12
Fragmentor voltage (V)	380
**Scan Source Parameter**	
Capillary voltage (V)	3000
Nozzle voltage (V)	300

References

1. Röhler K, Susset B, Grathwohl P (2023) Production of perfluoroalkyl acids (PFAAs) from precursors in contaminated agricultural soils: Batch and leaching experiments. Sci Total Environ 902:166555. doi:10.1016/j.scitotenv.2023.166555

2. Bugsel B, Bauer R, Herrmann F, Maier ME, Zwiener C (2022) LC-HRMS screening of per- and polyfluorinated alkyl substances (PFAS) in impregnated paper samples and contaminated soils. Anal Bioanal Chem 414 (3):1217-1225. doi:10.1007/s00216-021-03463-9
